# CUL4B facilitates HBV replication by promoting HBx stabilization

**DOI:** 10.20892/j.issn.2095-3941.2020.0468

**Published:** 2022-01-15

**Authors:** Haixia Shan, Bo Wang, Xiaodong Zhang, Hui Song, Xi Li, Yongxin Zou, Baichun Jiang, Huili Hu, Hao Dou, Changshun Shao, Lifen Gao, Chunhong Ma, Xiaoyun Yang, Xiaohong Liang, Yaoqin Gong

**Affiliations:** 1 Key Laboratory for Experimental Teratology of the Ministry of Education, Key Laboratory of Infection and Immunity of Shandong Province and Department of Immunology, School of Basic Medical Sciences, Shandong University, Jinan 250012, China; 2 Department of Laboratory Diagnosis, Cangzhou Combination of Traditional Chinese and Western Medicine Hospital of Hebei Province, Cangzhou 061000, China; 3 Division of Gastroenterology and Hepatology, Renji Hospital, School of Medicine, Shanghai Jiao Tong University, Shanghai Institute of Digestive Disease, Shanghai 200120, China; 4 Ministry of Education Key Laboratory of Experimental Teratology and Institute of Molecular Medicine and Genetics, School of Basic Medical Sciences, Shandong University, Jinan 250012, China; 5 Collaborative Innovation Center of Technology and Equipment for Biological Diagnosis and Therapy in Universities of Shandong, Jinan 250012, China; 6 Department of Gastroenterology, Qilu Hospital, Shandong University, Jinan 250012, China

**Keywords:** CUL4B, HBV, ubiquitination, HBx, HCC

## Abstract

**Objective:**

Hepatitis B virus (HBV) infection is a major public health problem worldwide. However, the regulatory mechanisms underlying HBV replication remain unclear. Cullin 4B-RING ubiquitin E3 ligase (CRL4B) is involved in regulating diverse physiological and pathophysiological processes. In our study, we aimed to explain the role of CUL4B in HBV infection.

**Methods:**

*Cul4b* transgenic mice or conditional knockout mice, as well as liver cell lines with CUL4B overexpression or knockdown, were used to assess the role of CUL4B in HBV replication. Immunoprecipitation assays and immunofluorescence staining were performed to study the interaction between CUL4B and HBx. Cycloheximide chase assays and *in vivo* ubiquitination assays were performed to evaluate the half-life and the ubiquitination status of HBx.

**Results:**

The hydrodynamics-based hepatitis B model in *Cul4b* transgenic or conditional knockout mice indicated that CUL4B promoted HBV replication (*P* < 0.05). Moreover, the overexpression or knockdown system in human liver cell lines validated that CUL4B increased HBV replication in an HBx-dependent manner. Importantly, immunoprecipitation assays and immunofluorescence staining showed an interaction between CUL4B and HBx. Furthermore, CUL4B upregulated HBx protein levels by inhibiting HBx ubiquitination and proteasomal degradation (*P* < 0.05). Finally, a positive correlation between CUL4B expression and HBV pgRNA level was observed in liver tissues from HBV-positive patients and HBV transgenic mice.

**Conclusions:**

CUL4B enhances HBV replication by interacting with HBx and disrupting its ubiquitin-dependent proteasomal degradation. CUL4B may therefore be a potential target for anti-HBV therapy.

## Introduction

Hepatitis B virus (HBV) infection remains a serious health problem. Despite the availability of an effective preventive vaccine, 257 million people worldwide are chronically infected with HBV and are at increased risk of developing liver cirrhosis and hepatocellular carcinoma (HCC). Each year, an estimated 600,000 people die from HBV-related liver diseases^1^.

HBV is a hepatotropic, non-cytopathic, small enveloped DNA virus. The encapsidated viral genome consists of a 3.2 kb partially double-stranded relaxed circular DNA (rcDNA)^2^. After viral entry into hepatocytes, the capsid dissociates, and the rcDNA genome translocates into the nucleus and is converted into a covalently closed circular DNA molecule. This molecule is then transcribed and produces pre-genomic RNA (pgRNA) and other subgenomic viral RNAs. HBV polymerase (Pol) then recognizes the 5′-ε signal in pgRNA and facilitates the encapsidation of pgRNA into a newly formed HBV capsid, wherein pgRNA is reverse transcribed into rcDNA. Mature nucleocapsids with HBV rcDNA are then released from the cells after being enveloped with HBs proteins or recycled to the closed circular DNA reservoir in the nucleus^3^.

A variety of viral and host factors contribute to regulating HBV replication. The ubiquitin-proteasome pathway of protein degradation is also involved in modulating the life cycle of HBV. Bandi et al.^4^ have reported that the proteasome inhibitor bortezomib (Velcade) dose-dependently inhibits HBV replication as well as viral RNA and protein expression. However, another proteasome inhibitor, MLN-273, has only a minor effect on wild-type HBV transgenic mice, but substantially enhances HBV replication in HBx-deficient HBV transgenic mice^5^. These findings indicate that the proteasome system might play complicated roles in HBV replication. However, similarly to other viral proteins, HBV viral proteins interact with or otherwise modulate the ubiquitin-proteasome activity, thus manipulating the cellular environment to the advantage of the virus and/or resulting in physiopathological changes in host cells^6^. The HBV X protein (HBx), well-known for its pleiotropic roles in HBV replication and its numerous binding partners in both the cytoplasm and nucleus^7^, has been shown to interact with several proteasome subunits, including PSMA7 and PSMC1, and to inhibit proteasome activation or the degradation of ubiquitinated proteins, thus facilitating viral persistence^8,9^.

CUL4B, a member of the Cullin 4 proteins, assembles Cullin 4B-Ring E3 ligases (CRL4B), together with DDB1 and ROC1^10^. Unlike CUL4A and other Cullin proteins, CUL4B mainly localizes to the nucleus, as directed by its unique N-terminal nuclear sequence, and mediates either polyubiquitination for proteasomal degradation of substrates or H2A monoubiquitination for epigenetic regulation^11^. Loss-of-function mutations in human CUL4B lead to X-linked mental retardation, and *Cul4b* knockout is embryonically lethal in mice^12^, thus indicating the important biological roles of the *Cul4b* gene. Accumulating data indicate that CRL4B displays pleiotropic functions in both physiologic (e.g., DNA replication licensing^13^, cell cycle regulation^14^, and metabolic homeostasis^15^) and pathologic (carcinogenesis^11,16,17^ and obesity^18^) milieux. In viral infection, CRL4B is hijacked by viral proteins, thus causing polyubiquitination and proteasomal degradation of host proteins. For example, HIV Vpr and Vpx bind with CRL4B, thus triggering the degradation of the DNA repair protein uracil-N-glycosylase 2 (UNG2), the anti-viral protein SAMHD1, and the human silencing hub complex HUSH, which promotes viral replication and HIV pathogenesis^19^. To date, no direct evidence has been reported regarding the role of CRL4B in HBV replication. However, crystallographic and functional analyses have revealed an interaction of HBx with UV-damaged DNA binding protein 1 (DDB1), an evolutionarily conserved adaptor protein for CUL4-RING E3 ubiquitin ligases^20^ that is required for HBx protein stability^21^ and has biological roles in viral promoter activation and cell cycle dysregulation^22,23^. Moreover, CRL4 is recruited by HBx and functions in the ubiquitination and degradation of the structural maintenance of chromosomes (SMC) complex proteins SMC5/6, thus restricting HBV replication by inhibiting HBV gene expression^24^. These findings suggest that CUL4B-RING E3 ligase might be involved in HBV replication.

Here, we provide the first reported evidence that CUL4B promotes HBV replication in an HBx-dependent manner. Interestingly, CUL4B protects HBx against proteasomal degradation. Moreover, CUL4B expression is positively correlated with the HBV replication level in HBV-positive liver tissues. Our findings thus reveal a novel mechanism underlying the involvement of CUL4B E3 ubiquitin ligase in HBV replication and shed light on a potential therapeutic target for HBV elimination.

## Materials and methods

### Clinical samples

HBV-positive liver tissues from patients with hepatic cavernous hemangioma or distant nontumor normal liver tissues from HBV-positive HCC patients were collected at Qilu Hospital and Shandong Provincial Hospital affiliated with Shandong University (Shandong, China) between October 30 2012 and August 31 2015. All patients were negative for HCV or HIV, had no history of heavy drinking, and did not receive any therapy before surgery. Informed consent was obtained from all patients before the study was performed, with the approval of Shandong University Medical Ethics Committee in accordance with the Declaration of Helsinki (approval No. 2011023). All tissues were stored at −80 °C for analysis of HBV pgRNA levels and CUL4B expression.

### Plasmids and cell lines

pcDNA3-HBV1.1, carrying 110% of HBV genotype C, pcDNA3-HBx-HA, and pcDNA3-Pol, containing C-terminal HA-tagged HBx and a polymerase gene fragment, were as described previously^25^. HBx-null HBV plasmids (ΔHBx) with a stop codon at position 7 of the HBx open reading frame, and polymerase-null HBV plasmids (ΔPol) with a frame-shift mutation involving deletion of the T nucleotide of the second ATG, and a point mutation of the first ATG to ACG in the polymerase open reading frame, which did not alter the encoded amino acid, were constructed with a PCR-based specific mutagenesis kit (TOYOBO, Shanghai, China). The human HCC cell lines HepG2 and SMMC7721, human embryonic kidney 293 (HEK293) cells, and the human cervical cancer cell line HeLa were purchased from the Shanghai Cell Collection (Chinese Academy of Sciences). These cells were maintained in DMEM (Gibco, Invitrogen) containing 10% fetal bovine serum, 100 units/mL penicillin, and 100 µg/mL streptomycin (Invitrogen, Beijing, China). Cells were grown under 5% CO_2_ at 37 °C. HEK293 or HeLa cell lines stably infected with CUL4B shRNA lentivirus were generated as described previously^13^. Transfections were performed with Lipofectamine 2000 (Invitrogen) according to the manufacturer’s instructions.

### Mice and hydrodynamic injection model

HBV complete genome (ayw subtype) transgenic Balb/c mice expressing high levels of HBV antigens and HBV DNA were purchased from the Transgenic Animal Central Laboratory (458 Hospital, Guang Zhou, China). The *Cul4b* transgenic CD1 mice were generated with the pEGFP-CUL4B construct. The *Cul4b* floxed mice were produced as described previously^11^. To generate mice with inducible *Cul4b* deletion, we crossed *Cul4b*-floxed mice with Mx1-Cre or Alb-Cre transgenic mice (The Jackson Laboratory)^26^. In the Mx1-Cre; Cul4b^flox/y^ mice, the ablation of *Cul4b* was achieved through 6 injections of poly(I:C) i.p. at 48 h intervals.

Six- to seven-week-old male *Cul4b* transgenic mice, Mx1-Cre; Cul4b^flox/y^ mice, Alb-Cre; Cul4b^flox/y^ mice or the corresponding control mice were hydrodynamically injected with pcDNA3-HBV1.1^27^. In brief, 50 µg of pcDNA3-HBV1.1 in phosphate-buffered saline (PBS) was intravenously injected into the anesthetized mice at a volume equivalent to 8% of the body weight within 5–8 s. Twenty-four hours later, the liver tissues were collected, and HBV pgRNA levels were examined.

All mice were housed in the Department of Genetics, Shandong University under pathogen-free conditions. All animal procedures were in compliance with national regulations and approved by the Animal Use Committee, Shandong University School of Medicine (approval No. 2011008).

### Immunoprecipitation

HEK293 cells that had been transfected with pcDNA3-HBx-HA for 48 h were washed with cold PBS; lysed with lysis buffer containing 1% NP-40, 50 mM Tris-HCl pH 7.4, 50 mM EDTA, 150 mM NaCl and a protease inhibitor cocktail for 30 min at 4 °C; and then centrifugated for 20 min at 12,000 g. The supernatants were collected and incubated with 2 µg of anti-CUL4B, anti-HA or normal rabbit/mouse immunoglobulin G (IgG) at 4 °C overnight. Protein A/G agarose beads (Santa Cruz Biotechnology) were added to the mixture and incubated for 6 h, then washed 6 times with cold PBS. The beads were eluted with 1% SDS, boiled 5 min, and subjected to SDS-PAGE followed by immunoblotting with specific primary and corresponding secondary antibodies. The immunodetection was performed with ECL.

### *In vivo* ubiquitination assays

HEK293 cells were cotransfected with HA-tagged HBx and CUL4B siRNA or negative control siRNA. Forty-eight hours later, cells were treated with MG132 for 6 h. The cell lysates were immunoprecipitated with anti-HA antibody and then subjected to Western blot with antibodies specific to Ub and HA.

### Statistical analysis

Data are reported as mean values ± *SEM*. Cell experiments were performed in triplicate, and a minimum of 3 independent experiments were evaluated. GraphPad Prism (GraphPad Software, San Diego, CA, USA) was used for data analysis. The statistical significance of differences between groups was determined with Student’s t test. Spearman correlation analysis was performed between CUL4B expression and HBV replication markers. *P* values < 0.05 were considered significant.

## Results

### CUL4B promotes HBV replication both *in vivo* and *in vitro*

**Figure 1:**
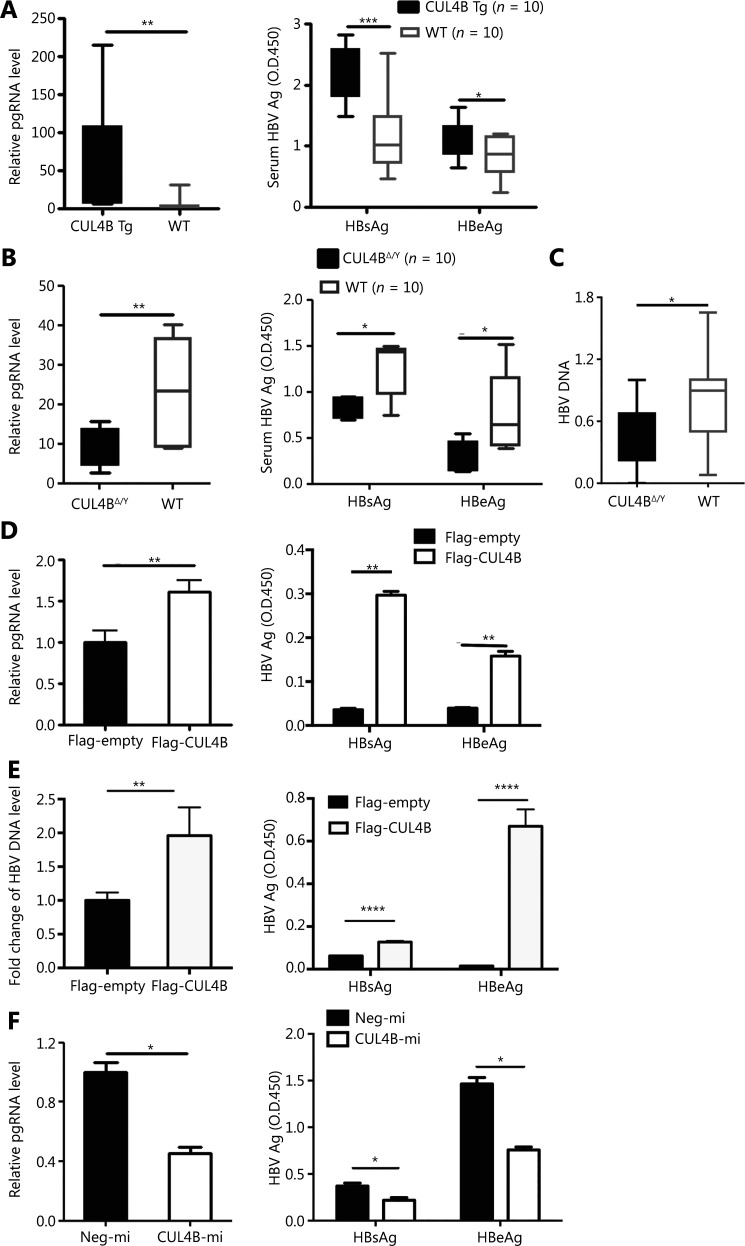
CUL4B enhances HBV replication both *in vivo* and *in vitro*. (A) pcDNA3-HBV1.1 plasmid was hydrodynamically injected into CUL4B Tg mice or WT control mice. Twenty-four hours later, the mice were sacrificed, and the serum or liver tissues were used for monitoring of HBV replication. The pgRNA levels (left panel) in liver tissues were detected by qPCR. Serum HBV antigen levels (right panel) were detected by ELISA. (B) Mx1-Cre; CUL4B^flox/Y^ mice (CUL4B^Δ/Y^) or Mx1-Cre; CUL4B^+/Y^ mice (WT) were peritoneally injected with PIPC (300 µg per mouse) at 48 h intervals for 6 times. Five days after the last PIPC injection, pcDNA3-HBV1.1 plasmid was hydrodynamically injected into CUL4B^Δ/Y^ mice or control mice. Twenty-four hours later, the mice were sacrificed, and the pgRNA (left panel) in the liver (middle panel) and the HBsAg/HBeAg level in the serum (right panel) were detected. (C) Alb-Cre; CUL4B^flox/Y^ mice (CUL4B^Δ/Y^) or Alb-Cre; CUL4B^+/Y^ mice (WT) were hydrodynamically injected with pcDNA3-HBV1.1 plasmid. Twenty-four hours later, the mice were sacrificed, and HBV DNA in liver tissues was detected by qPCR. (D) pcDNA3-HBV1.1 plus Flag-CUL4B or Flag-empty were transfected into HepG2 cells; 48 h later, the pgRNA levels (left panel) in HepG2 cells were detected by qPCR. HBV antigen levels in cell supernatants (right panel) were detected by ELISA. (E) pcDNA3-HBV1.1 plus Flag-CUL4B or Flag-empty were transfected into Huh7 cells; 48 h later, HBV DNA levels (left panel) in Huh7 cells were detected by qPCR. HBV antigen levels in cell supernatants (right panel) were detected by ELISA. (F) pcDNA3-HBV1.1 plus CUL4B-miRNA or Neg-miRNA was transfected into HepG2 cells; 48 h later, pgRNA levels (left panel) in HepG2 cells were detected by qPCR. HBV antigen levels in cell supernatants (right panel) were detected by ELISA. The data in panels A–C are the mean *± SEM*, with each sample value from an individual mouse. The data in panels D and E are the mean ± *SD* of 3 independent experiments, and Student’s *t*-test was performed (*P < 0.05; **P < 0.01; ***P < 0.001; ****P < 0.0001).

To understand the role of CUL4B in regulating HBV replication, we generated hydrodynamic-based HBV infection models in both *Cul4b* transgenic mice (**Supplementary Figure S1A**) and *Cul4b* conditional knockout mice (**Supplementary Figure S1B**). After hydrodynamic tail-vein injection of pcDNA3-HBV1.1, *Cul4b* transgenic mice displayed higher HBV pgRNA (*P* < 0.01) and serum HBsAg (*P* < 0.001) and HBeAg (*P* < 0.05) levels than those in wild type mice (**Figure 1A**). Accordingly, Mx1-Cre; *Cul4b*^flox/Y^ mice treated with polyinosinic-polycytidylic acid to induce ablated CUL4B expression in the liver (**Supplementary Figure S1B**) had lower levels of pgRNA (*P* < 0.01) and serum HBV antigen (*P* < 0.01) than wild type mice (**Figure 1B**). In line with these findings, lower serum HBV DNA levels were also observed in Alb-Cre; *Cul4b*^flox/Y^ mice than in wild type mice (**Figure 1C**) (*P* < 0.01). These data revealed that CUL4B promotes HBV replication *in vivo*.

To further validate CUL4B-mediated promotion of HBV replication, we next performed overexpression and loss-of function experiments in HCC cell lines. CUL4B overexpression, as compared with the control, significantly upregulated the levels of HBV pgRNA (*P* < 0.01) and secreted HBV antigens (*P* < 0.01) in HepG2 cells (**Supplementary Figure S2A, Figure 1D**). Accordingly, CUL4B overexpression significantly upregulated the levels of HBV DNA (*P* < 0.01) and secreted HBV antigens (*P* < 0.0001) in Huh7 cells (**Figure 1E**). In contrast, a specific miRNA construct against CUL4B in HepG2 cells led to markedly decreased levels of HBV replication markers (**Supplementary Figure S2B, Figure 1F**) (*P* < 0.01). Similar results were further verified in BEL7402 and HepG2.2.15 cells (**Supplementary Figure S3**). Collectively, our *in vivo* and *in vitro* data clearly showed that CUL4B increases HBV replication.

### HBx is responsible for optimal CUL4B-promoted HBV replication

**Figure 2:**
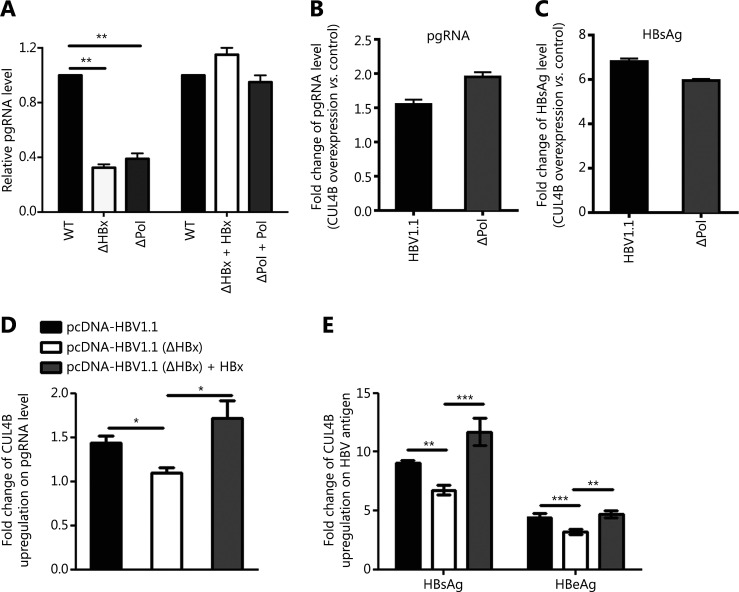
HBx is required for CUL4B-mediated enhancement of HBV replication. (A) pcDNA3-HBV1.1, pcDNA3-HBV1.1(ΔHBx), pcDNA3-HBV1.1(ΔHBx) plus pcDNA3-HBx, pcDNA3-HBV1.1(ΔPol), or pcDNA3-HBV1.1(ΔPol) plus pcDNA3-Pol was transfected into HepG2 cells. Forty-eight hours later, pgRNA levels were detected by qPCR. (B, C) HepG2 cells were cotransfected with pcDNA3-HBV1.1 or pcDNA3-HBV1.1(ΔPol) together with Flag-CUL4B or Flag-empty. pgRNA levels were detected by qPCR (B), and HBsAg was detected by ELISA (C). (D, E) pcDNA3-HBV1.1, pcDNA3-HBV1.1(ΔHBx), and pcDNA3-HBV1.1(ΔHBx) plus pcDNA3-HBx were cotransfected into HepG2 cells together with Flag-CUL4B or Flag-empty. After transfection for 48 h, pgRNA (D) and HBV antigen levels in cell supernatant (E) were detected. The fold change in CUL4B upregulation was calculated for each group relative to Flag-empty controls. All data are shown as the mean ± *SD* of 3 independent experiments, and Student’s *t*-test was performed (*P < 0.05; **P < 0.01; ***P < 0.001).

To explore the mechanisms through which CUL4B promotes HBV replication, we first investigated whether HBV viral proteins might be involved in this regulatory process. For this purpose, HBx-null or Pol-null HBV plasmids were cotransfected with CUL4B expression vector into HepG2 cells, and the effects of CUL4B on the replication of these different HBV replicons were analyzed. As expected, HBx-null or Pol-null HBV plasmids resulted in a marked decrease in virus replication, which was restored to wild type levels by the corresponding HBx or polymerase expression plasmids (**Figure 2A**) (*P* < 0.01). Interestingly, although CUL4B upregulated wild type and Pol-null HBV replication to similar levels (**Figure 2B and 2C**), this regulatory effect significantly decreased for the HBx-null HBV replication, thus resulting in comparable levels of pgRNA and secreted HBV antigens to those in control cells; in contrast, the ectopic HBx expression plasmid rescued the CUL4B-mediated promotion of HBx-null HBV replication (**Figure 2D and 2E**) (*P* < 0.01). Together, these results suggest that HBx is required for CUL4B-mediated regulation of HBV replication.

### HBx is physically associated with CUL4B-RING E3 ligase

**Figure 3:**
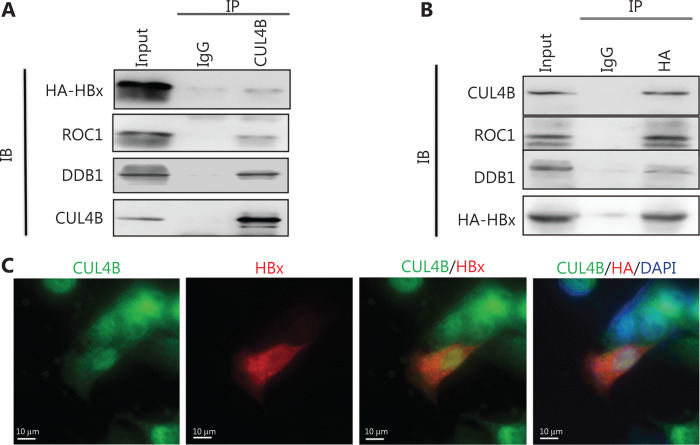
HBx interacts with the CUL4B-DDB1-Roc1 complex. (A, B) pcDNA3-HBx-HA was transfected into HEK293 cells; 24 h later, whole cell lysates were immunoprecipitated with antibodies against CUL4B (A) or HA (B). Immunocomplexes were then immunoblotted with antibodies against the indicated proteins. IgG served as the negative control. (C) HepG2 cells were transfected with pcDNA3-HBx-HA for 48 h. Immunofluorescence staining was used to detect the interaction between HBx and CUL4B.

DDB1 has been found to be essential to the role of HBx in HBV replication, through interaction with HBx^21^. Interestingly, DDB1 functions as an evolutionarily conserved adaptor protein for CUL4-RING E3 ubiquitin ligases^20^. Thus, we hypothesized that HBx might be physically associated with CUL4B-RING E3 ligase. To address this possibility, we subjected total proteins from HEK293 cells transfected with HA-tagged HBx to coimmunoprecipitation experiments. CUL4B efficiently coimmunoprecipitated with HBx as well as DDB1 and ROC1 (**Figure 3A**), and vice versa (**Figure 3B**). To provide further support for the association between CUL4B and HBx, we subjected HepG2 cells transfected with HA-tagged HBx expression plasmid to fixing and staining with antibodies recognizing CUL4B and HA tag. As shown in **Figure 3C**, the signals representing CUL4B were predominantly distributed in the nuclei in HepG2 cells and colocalized with the HBx signal. We also observed colocalization of CUL4B and HBx in human liver tissues (**Supplementary Figure S4**). Collectively, these results support the physical association of HBx with CUL4B-RING E3 ligase.

### CUL4B promotes the accumulation of HBx protein

**Figure 4:**
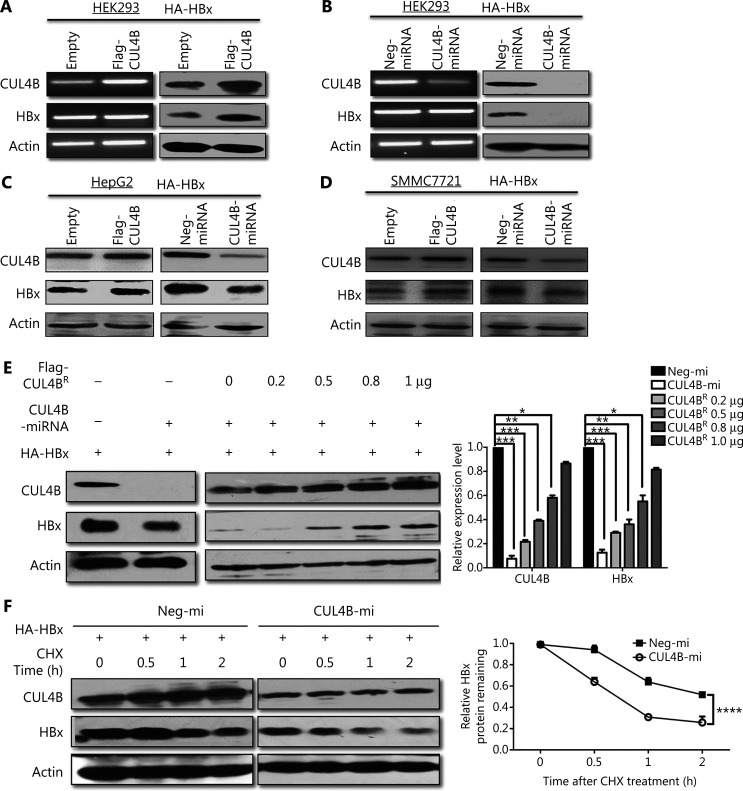
CUL4B upregulates the HBx protein level. (A, B) pcDNA3-HBx was transfected into HEK293 cells together with Flag-CUL4B, or CUL4B-miRNA, or control plasmid; 24 h later, CUL4B or HBx expression was detected by RT-PCR or Western blot. (C, D) HepG2 or SMMC7721 cells were cotransfected with pcDNA3-HBx plus Flag-CUL4B, or CUL4B-miRNA, or control plasmid. CUL4B or HBx expression was detected by Western blot. (E) HepG2.2.15 cells were transfected with Neg-miRNA or CUL4B-miRNA; 48 h later, CUL4B or HBx expression was detected by Western blot. (F) HeLa cells stably expressing Neg-miRNA or CUL4B-miRNA were transfected with HBx alone, or HBx plus CUL4B-miRNA rescue plasmid Flag-CUL4B^R^; 24 h later, CUL4B or HBx expression was detected by Western blot. (G) A representative CHX chase analysis of HBx protein degradation in HEK293 cells. HEK293 cells stably expressing Neg-miRNA or CUL4B-miRNA were transfected with HBx for 24 h. Then, the cells were treated with CHX for the indicated period, and the HBx expression was detected by Western blot (left panel). Relative HBx protein remaining was calculated according to the band density of HBx; β-actin served as an internal control (right panel). Student’s *t*-test was performed (*P < 0.05; **P < 0.01; ***P < 0.001; ****P < 0.0001).

To further explore the functional connection between CUL4B and HBx, we next investigated the potential regulation of CUL4B in HBx expression. HA-tagged HBx and CUL4B expression plasmid or empty vector were cotransfected into HEK293 cells. Although CUL4B showed no detectable effect on HBx mRNA levels, the protein level of HBx was dramatically upregulated in HEK293 cells with CUL4B overexpression (**Figure 4A and Supplementary Figure S5A**). In contrast, CUL4B knockdown significantly decreased HBx protein (**Figure 4B, Supplementary Figure S5B**). Moreover, similar results were found in 3 HCC cell lines, HepG2 (**Figure 4C and Supplementary Figure S5C**), SMMC7721 (**Figure 4D and Supplementary Figure S5D**), and HepG2.2.15 (**Supplementary Figure S5E**), thus validating that CUL4B promotes the accumulation of HBx protein. To further confirm this effect of CUL4B on HBx, we transiently transfected different doses of Flag-tagged, full-length RNAi-insensitive CUL4B expression vector into CUL4B-miRNA stably transfected HeLa cells. As shown in **Figure 4E**, the decreased HBx protein in CUL4B knockdown cells was rescued by the RNAi-resistant CUL4B expression vector in a dose-dependent manner (*P* < 0.05). These results supported the argument that CUL4B maintains HBx protein expression.

To test the possibility that CUL4B might regulate HBx degradation, we measured the half-life of HBx protein. HEK293 cells stably expressing Neg-miRNA or CUL4B-miRNA were transfected with HBx and treated with cycloheximide (CHX) for the indicated intervals. Silencing of CUL4B resulted in a significant decrease in the half-life of HBx (**Figure 4F**) (*P* < 0.00001), thus further supporting that CUL4B promotes the accumulation of HBx.

### CUL4B inhibits the ubiquitination and proteasomal degradation of HBx

**Figure 5:**
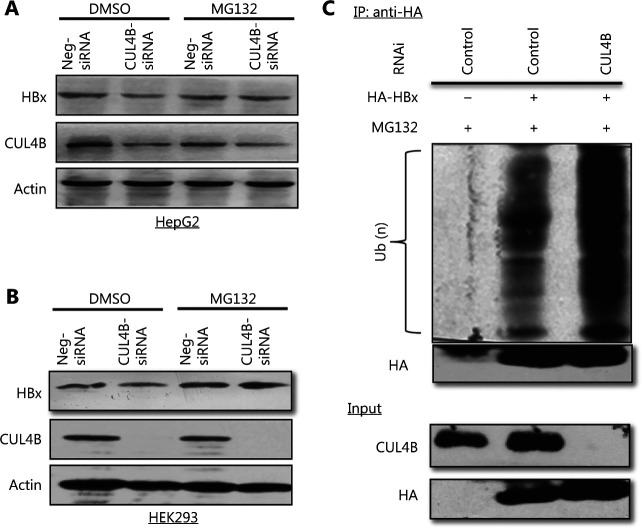
CUL4B inhibits the ubiquitination and proteasomal degradation of HBx. (A, B) HepG2 or HEK293 cells were cotransfected with pcDNA3-HBx plus CUL4B-siRNA or negative control siRNA; 18 h after transfection, cells were exposed to 50 µg/mL MG132 or DMSO. Then, 6 h later, CUL4B or HBx expression was detected by Western blot. (C) HEK293 cells stably expressing Neg-miRNA or CUL4B-miRNA were transfected with HBx for 16 h. Then the cells were treated with 50 µg/mL MG132 for another 6 h. Cell lysates were immunoprecipitated with anti-HA antibody and immunoblotted with anti-Ub antibody to detect the ubiquitinated HBx protein.

HBx is a protein with rapid turnover, which is finely regulated by proteasome-mediated degradation through ubiquitination^8^. We therefore sought to determine whether CUL4B might affect the ubiquitination and proteasomal degradation of HBx. To test this possibility, we transfected CUL4B knockdown or control HepG2 cells, with HA-tagged HBx expression vector, then treated them with MG132, an inhibitor of the 26S proteasome. Although CUL4B knockdown led to a severe decrease in HBx protein in the DMSO-treated group, MG132 treatment substantially mitigated this inhibitory effect (**Figure 5A and Supplementary Figure S6**). Similar results were obtained in HEK293 cells (**Figure 5B and Supplementary Figure S6**). These data support that CUL4B regulates HBx expression in a proteasomal-degradation dependent manner.

We next investigated whether CUL4B might also affect HBx ubiquitination. HEK293 cells were transfected with a HA-tagged HBx expression construct together with a construct for CUL4B knockdown. The cell lysates were immunoprecipitated with anti-HA antibody and immunoblotted with anti-Ub antibody to detect ubiquitinated HBx proteins. As shown in **Figure 5C**, HBx ubiquitination was significantly higher in cells with CUL4B knockdown (**Figure 5C, lane 3**) than in control cells. Collectively, these results further supported our hypothesis that CUL4B protects HBx expression from ubiquitination and proteasomal degradation.

### CUL4B expression is positively correlated with HBV replication in human and mouse livers

**Figure 6:**
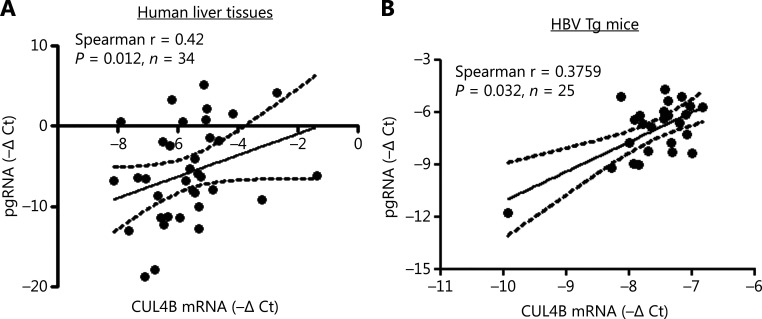
The positive correlation of CUL4B mRNA with HBV replication in human and mouse livers. (A, B) HBV pgRNA and CUL4B mRNA in HBV-positive human liver tissues or in HBV Tg mouse liver tissues were detected with real-time PCR. The correlation of HBV pgRNA levels with CUL4B mRNA expression in HBV-positive human liver tissues (A) or in HBV Tg mouse liver tissues (B) was statistically analyzed.

To further demonstrate CUL4B regulation of HBV replication, we analyzed the relationship between CUL4B expression and HBV pgRNA level in liver tissues from patients with chronic HBV and from HBV transgenic mice. CUL4B mRNA in HBV-positive human liver tissues positively correlated with pgRNA levels (**Figure 6A**) (*P* < 0.01). Moreover, a positive correlation between CUL4B mRNA and HBV pgRNA levels was found in liver tissues from HBV transgenic mice (**Figure 6B**) (*P* < 0.01). Collectively, these data further suggested that CUL4B is indeed involved in regulating HBV replication.

## Discussion

A wide array of viral and host factors contribute to the regulation of HBV replication. Exploration of the novel regulatory mechanisms and development of the potential therapeutic targets for HBV-related diseases are urgently needed. In this report, we first identified the effect of CUL4B E3 ligase on HBV replication and found that suppression of CUL4B had potent antiviral effects. Chronic HBV infection is a high-risk factor for human HCC^28^. Thus, CUL4B’s promotion of HBV replication might also be involved in the development of HBV-associated HCC. Indeed, our previous study has shown that CUL4B also promotes the malignancy of HCC by upregulating Wnt/β-catenin signaling^29^. In mice, overexpression of CUL4B strongly promotes spontaneous and DEN-induced hepatocarcinogenesis^30^. Thus, inhibition of CUL4B might be a potential therapeutic strategy for HCC treatment by effectively suppressing both HBV replication and HCC malignancy.

CUL4B has recently been reported to have an interchangeable role with that of CUL4A in HIV-induced cell cycle arrest and depletion of the anti-viral protein SAMHD1^19^. Although several studies have revealed that DDB1, the only known adaptor for CUL4 E3 ubiquitin ligases^20^, is required for maximal HBV replication through association with HBx, little is known about the exact role of the scaffold protein CUL4B in this process. In this work, results from both *Cul4b* transgenic or conditional knockout mice and HBV-infected cell lines verified that CUL4B promotes HBV replication. Moreover, we demonstrated the potential correlation between CUL4B expression and pgRNA levels in liver tissues from HBV-positive patients and HBV transgenic mouse liver tissues. These findings extend understanding of the relationship between CRL4B and HBV. In addition, although CUL4B and CUL4A share several functional similarities in certain biological milieux (e.g., HIV infection), owing to their 80% amino acid sequence identity^10^, the differences in the N-terminal protein sequences and subcellular distribution confer functional specificity. Whether CUL4A and CUL4B play redundant or complementary roles in HBV regulation is worthy of further study.

HBx, a regulatory viral protein, is well conserved among mammalian hepadnaviruses, thus suggesting an important biological function. Several studies have found that HBx protein is essential for the viral replication process^31,32^, mainly through promoting viral gene expression^33^. Here, we validated that HBx, but not polymerase, is indispensable for CUL4B regulation of HBV replication. CUL4B had no clear effects on HBx-null HBV replication. Immunoprecipitation assays and immunofluorescence staining further indicated the interaction of CUL4B with HBx. Given that DDB1 is a conserved binding partner of HBx^20^, CUL4B-RING ligase together with HBx might plausibly form a functional complex, and the integrity of this complex may be important for the efficient replication of HBV.

An important question is what biological functions are executed by the CRL4B ligase and HBx complex. We first analyzed the effect of CUL4B on HBx and found that CUL4B upregulates HBx protein expression by protecting it from uniquitination and proteasomal degradation. These results suggest that CUL4B determines the accumulation of HBx, thereby facilitating maximal HBV replication^31–33^. Nevertheless, the detailed mechanisms underlying how CUL4B inhibits HBx ubiquitination and proteasomal degradation remain to be determined. Several molecules have been reported to control or regulate the degradation of HBx. Siah-1 is one known E3 ligase that directly mediates HBx poly-ubiquitination and proteasomal degradation^34^. In addition, both Hdj1, a human Hsp40/DnaJ chaperone protein, and Id-1, a member of the HLH protein family, have been found to facilitate the proteasomal degradation of HBx^35,36^. Whether CUL4B E3 ligase disrupts HBx ubiquitination by competing for HBx binding or by modulating the function of these regulatory molecules must be studied further.

## Conclusions

**Figure 7:**
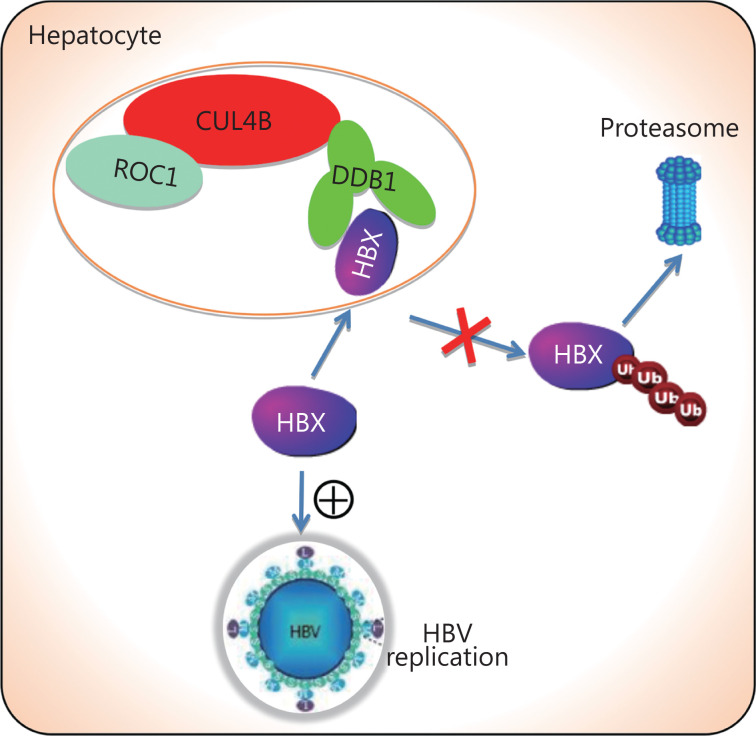
CUL4B promotes HBV replication *via* inhibition of HBV ubiquitination and degradation. Cullin 4B-RING ubiquitin E3 ligase interacts with HBx and inhibits its ubiquitin-dependent proteasomal degradation, thus facilitating HBV replication.

In summary, our study revealed that CUL4B enhances HBV replication by interacting with HBx and disrupting its ubiquitin-dependent proteasomal degradation, thus providing a molecular basis for the interplay between HBV and the host ubiquitin-proteasome system (**Figure 7**). Our data indicate that CUL4B stimulates HBV replication, thus supporting the potential of CUL4B as a target in anti-viral therapy.
