# Supplementary material for: CUL4B facilitates HBV replication by promoting HBx stabilization
Source: Cancer Biol Med. 2022 Jan 15;19(1):120–31. doi: 10.20892/j.issn.2095-3941.2020.0468 (PMC8763003; doi:10.20892/j.issn.2095-3941.2020.0468)
Supplement: Supplementary file 1 [file cbm-19-120-s001.pdf]

Supplementary materials

Materials and methods

Plasmids and siRNAs

A plasmid carrying 110% of hepatitis B virus (HBV) genome (pcDNA3-HBV1.1, subtype adr) and pcDNA3-HBx-HA, containing HA-tagged *HBx* gene, were described previously[25]. HBx-null HBV construct with a stop codon for amino acid 7 of HBx [pcDNA3-HBV1.1(ΔHBx)] and polymerase-null HBV construct with a point mutation at the initiation codon (ATG-ACG) [pcDNA3-HBV1.1(ΔPol)] were generated by PCR amplification using the KOD-Plus-Mutagenesis Kit (TOYOBO, Shanghai, China) with the primers described in **Supplementary Table S1**. HA-tagged *polymerase* gene fragment was amplified with pcDNA3-HBV1.1 using the primers in **Supplementary Table S1** and cloned into pcDNA3 with the restriction enzymes Kpn I and EcoR I (pcDNA3-Pol-HA). The pCMV-Tag2B-CUL4B, pCMV-Tag2B-CUL4B^R (RNAi-resistant CUL4B vector), and CUL4B-specific RNAi expression vector miCUL4B as well as their corresponding control plasmids were described previously[13].

The siRNAs were synthesized by (GenePharma, Shanghai, China) Co. Ltd and the targeted sequences were (sense sequences): *CUL4B*: 5′-CCACCCAGAAGTCATTA ATTT-3′; DDB1: 5′-GCGATAATAAAGAACTCAATT-3′; ROC1: 5′-GAAGCGCT TTGAAGTGAAATT-3′.

Detection of HBV antigens

The concentrations of HBsAg and HBeAg in cell culture supernatant or mouse sera were measured by using ELISA kits (Lizhu Co, Shenzhen, China) according to the manufacturer's instructions. The absorbance at a 450-nm wavelength was measured by a micro-ELISA reader.

RNA isolation and PCR

Total RNA was extracted from transfected cells or liver tissues using TRIzol reagent (Invitrogen, Shanghai, China) according to the manufacturer's instructions. The RNA was treated with RNase-free DNase (Thermo Fisher Scientific, Shanghai, China) and reversely transcribed into cDNA with RevertAid M-MuLV-RT (Thermo Fisher Scientific, Shanghai, China). The expression of CUL4B, pgRNA, and HBx was determined by real-time PCR with SYBR Green Master Mix (TOYOBO, Shanghai, China) or by regular PCR with Taq PCR Mastermix (Tangen, Beijing, China) according to the manufacturer's instructions, and the human or mouse β-actin was used as an internal control. All primer sequences are listed in **Supplementary Table S1**.

Western blot

The protein samples were lysed from transfected cells with CelLytic™ Cell Lysis Reagent (Sigma, Shanghai, China) supplemented with a protease inhibitor phenylmethylsulfonyl fluoride (PMSF) (Sigma, Shanghai, China). The cell lysates

Table S1 Primers used in vector construction and PCR assay

	Forward	Reverse
pcDNA3-HBV1.1(ΔHBx)	5′-CTAACTGGATCCTGCGCGGGACGTCCTTTG-3′	5′-CAGCACACCCGAGCAGCCATGGAAAGGAGG-3′
pcDNA3-HBV1.1(ΔPol)	5′-ACGCCCCTATCTTATCAACACTTCCG-3′	5′-TTGGTGGTCTGTAAGCAGGAGGAGTG-3′
pcDNA3-Pol-HA	5′-CCGGGTACCATGCCCCTATCTTATCAACACTTC-3′	5′-CCGGAATTCTAAGCGTAGTCTGGTACGTCGTAAGGG TACGGTGGTCTCCATGC-3′
pgRNA	5′-CTCAATCTCGGGAATCTCAATGT-3′	5′-AGGATAGAACCTAGCAGGCATAAT-3′
HBx	5′-TCCTTTGTCTACGTCCCG-3′	5′-TAATCTCCTCCCCCAACTCCTC-3′
Human CUL4B	5′-GCAACTGGAATAGAGGATGGA-3′	5′-TCTTTCTGTAGTGCTTGCTTGT-3′
Mouse CUL4B	5′-AGTGTCTGCCCAGGCACTT-3′	5′-GATTCCTCAGCCATTTTCGTAT-3′
Human β-actin	5′-AGTTGCGTTACACCCTTTC-3′	5′-CCTTCACCGTTCCAGTTT-3′
Mouse β-actin	5′-TGCGTGACATCAAAGAGAAG-3′	5′-TCCATACCCAAGAAGGAAGG-3′

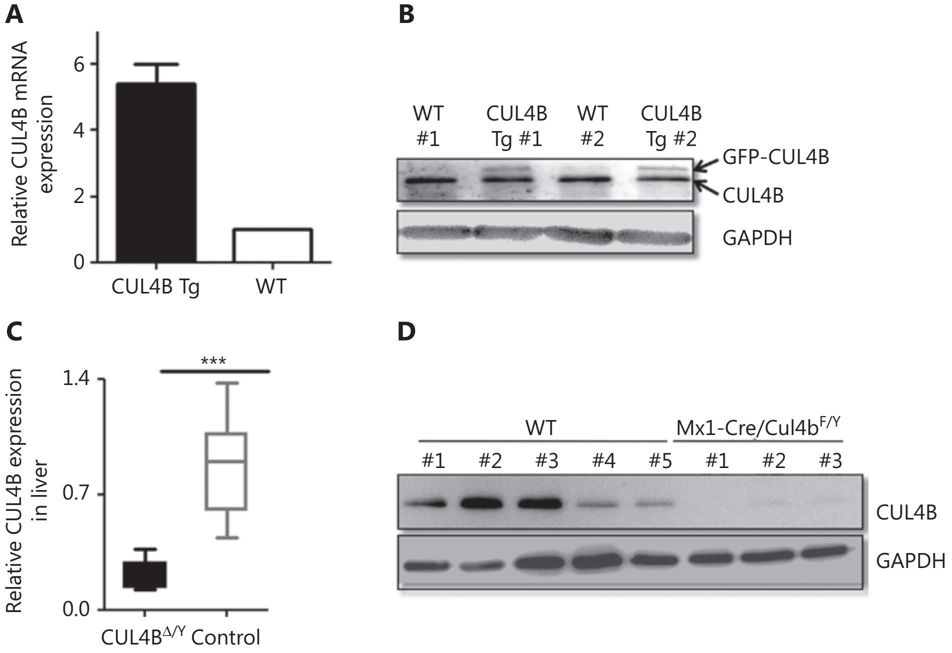

Figure S1 Related to **Figure 1**: CUL4B enhances hepatitis B virus (HBV) replication both *in vivo* and *in vitro*. (A–D) pcDNA3-HBV1.1 plasmid was hydrodynamically injected into CUL4B Tg mice (A, B), Mx1-Cre; CUL4B$^{flox/Y}$ mice (CUL4B$^{\Delta/Y}$) (pretreated with 300 μg PIPC per mouse) (C, D), or WT control mice. Twenty-four hours later, mice were sacrificed, Quantitative PCR (A, C) and Western blot (B, D) were performed to detect CUL4B mRNA and protein expression in liver. β-Actin was used as an internal control. Error bars represent mean ± *SD* of three independent experiments. (****P* < 0.001).

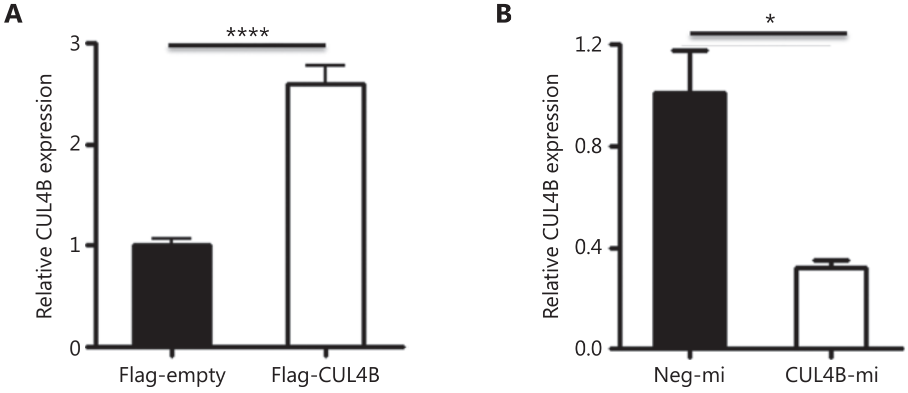

Figure S2 Related to **Figure 1**: CUL4B enhances hepatitis B virus (HBV) replication both *in vivo* and *in vitro*. (A, B) pcDNA3-HBV1.1 plus Flag-CUL4B (A), CUL4B-miRNA (B), or control plasmids were transfected into HepG2 cells. Forty-eight hours later, CUL4B mRNA expression in HepG2 cells was detected by qPCR. Error bars represent mean ± *SD* of three independent experiments. (**P* < 0.05; *****P* < 0.0001).

were incubated in ice for 30 min, and then centrifugated for 20 min at 12,000 rpm. The supernatants were collected, separated by sodium sulfate polyacrylamide gel electrophoresis (SDS-PAGE), and transferred on polyvinylidene difluoride (PVDF) membranes. After being blocked with 5% fat-free dry milk, the membranes were incubated with the specific antibodies against CUL4B (Sigma, Shanghai, China), Ub (BD Pharmingen™, Shanghai, China), anti-HA Tag (Abcam, Shanghai, China), anti-DDB1 (Santa Cruz Biotechnology, Dallas, Texas, USA), anti-ROC1 (Abcam, Shanghai, China), and β-actin (Sigma, Shanghai, China), respectively. The bound antibodies were detected with horseradish peroxidase

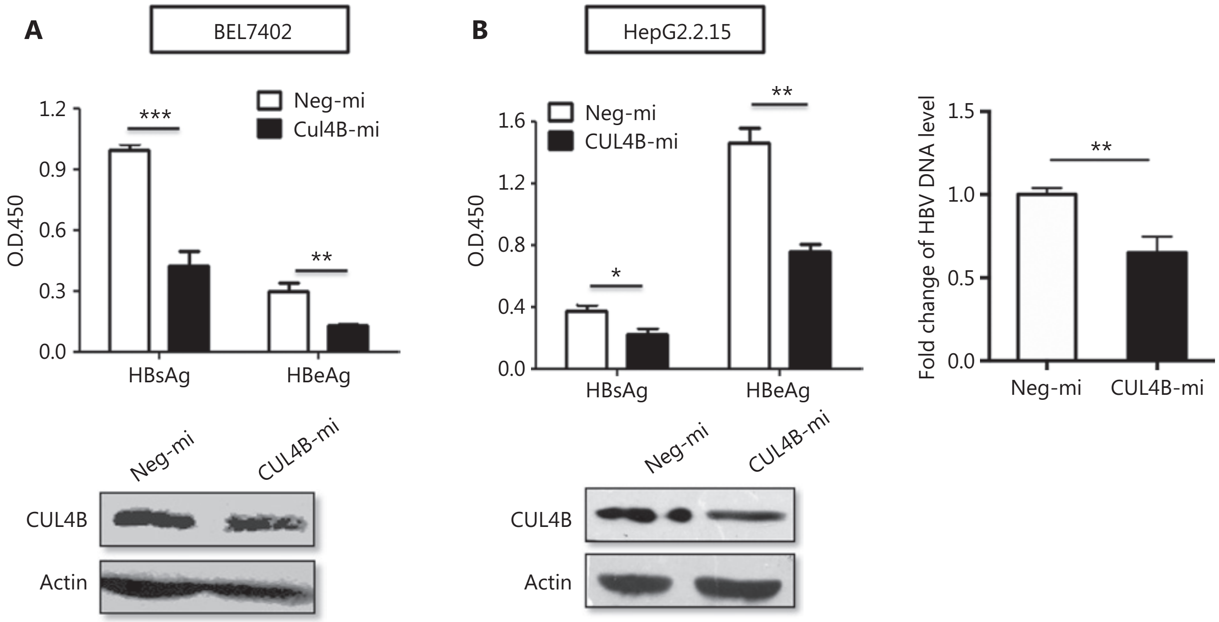

Figure S3 Related to **Figure 1**: CUL4B enhances hepatitis B virus (HBV) replication both *in vivo* and *in vitro*. BEL7402 cells were cotransfected with pcDNA3-HBV1.1 plus CUL4B-miRNA or Neg-miRNA. HepG2.2.15 cells, harboring four copies of HBV DNA and expressing all viral proteins, were transfected with CUL4B-miRNA or Neg-miRNA. Forty-eight hours later, HBV antigen levels in cell supernatant from BEL7402 (A) or HepG2.2.15 cells (B) were detected by ELISA (upper panel). HBV DNA level in HepG2.2.15 cells was detected by qPCR. (B) CUL4B protein expression was detected by Western blot (lower panel). Error bars represent mean ± *SD* of three independent experiments. (**P* < 0.05; ***P* < 0.01; ****P* < 0.001).

(HRP)-conjugated secondary antibodies and visualized using Enhanced Western Lightning Chemiluminescence Reagent (Amersham Biosciences, Piscataway, NJ, USA). The relative levels of target proteins to the control β-actin were determined by densitometry analysis using ImageJ software (National Institutes of Health, Bethesda, Maryland, USA).

Immunofluorescence

HeLa cells were transfected with pcDNA3-HBx-HA. Twenty-four hours later, cells were washed with Tris-buffered saline (TBS), fixed with 4% paraformaldehyde for 10 min at room temperature, and permeabilized with 0.2% Triton X-100 for 10 min. After blocking with 10% goat serum in phosphate-buffered saline (PBS) dilution for 15 min at 37 °C, cells were incubated with the mouse anti-HA (Abcam, Shanghai, China) and the rabbit anti-CUL4B (Sigma, Shanghai, China) antibodies at 4 °C overnight. Cells were washed with TBS and incubated for 60 min at 37 °C with rhodamine-conjugated goat anti-rabbit and fluorescein isothiocyanate (FITC)-conjugated rat anti-mouse secondary antibody (Jackson ImmunoResearch Laboratories, Inc. West Baltimore Pike West Grove, Pennsylvania, USA). After washing with TBS 4 times, cells were stained with DAPI (Sigma, Shanghai, China) for 15 min at 37 °C and further observed using fluorescence microscopy (Olympus, Shinjuku, Japan).

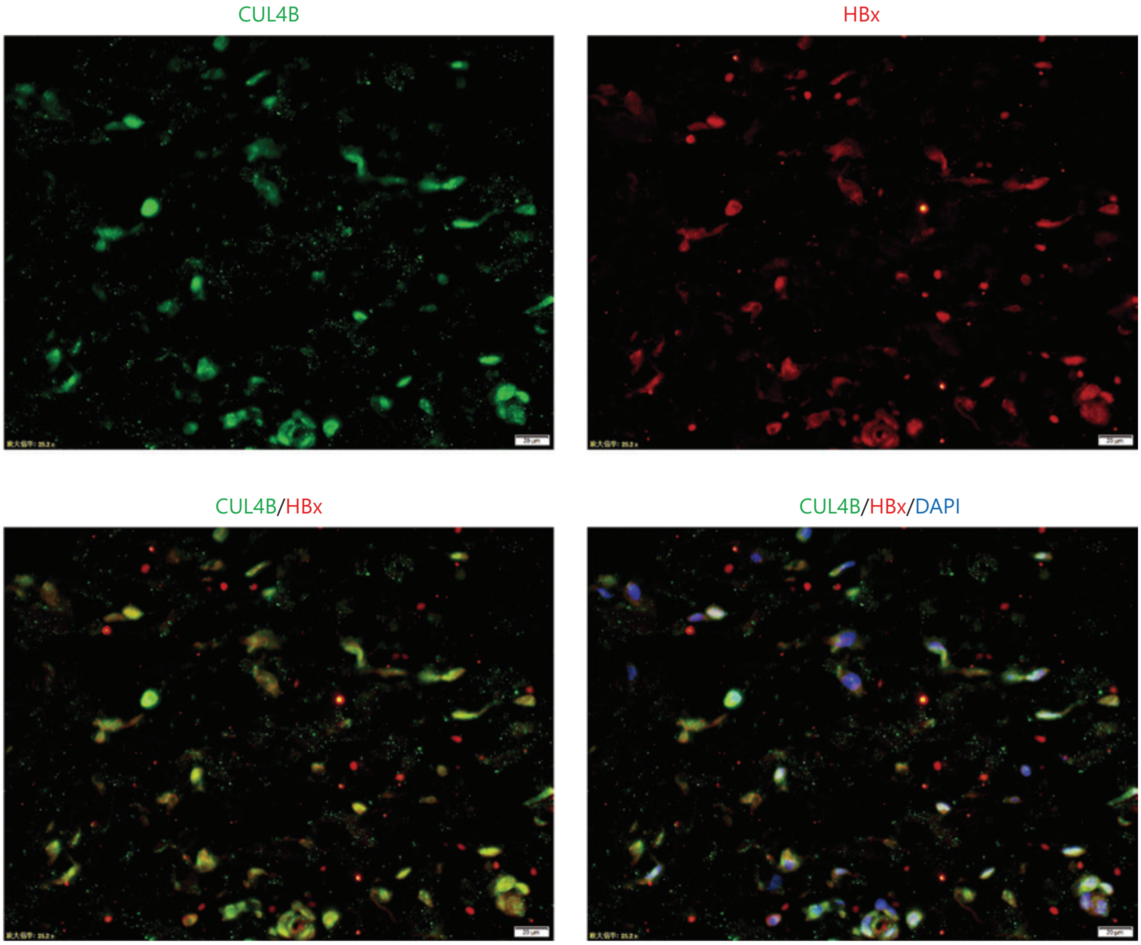

Figure S4 Related to **Figure 3**: HBx is physically associated with CUL4B in human liver tissue. The expression of CUL4B and HBx was detected by immunofluorescence staining in human liver tissues.

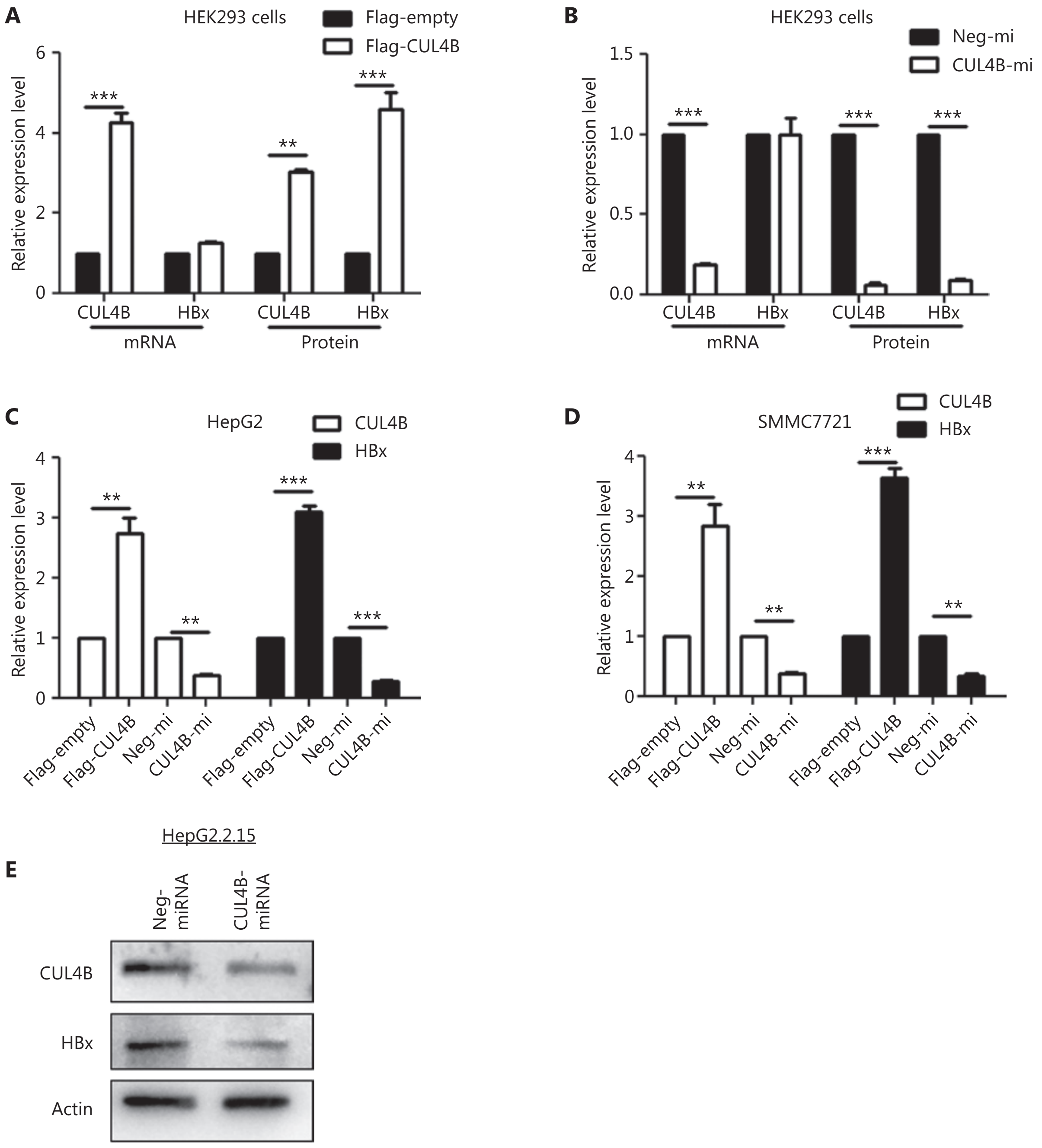

Figure S5 Related to **Figure 4**: CUL4B promotes the accumulation of HBx protein. Relative CUL4B or HBx mRNA or protein level was calculated as the ratio of band density relative to β-actin as an internal control in HEK293 (A, B), HepG2 (C), or SMMC7721 cells (D). (E) HepG2.2.15 cells were transfected with Neg-miRNA or CUL4B-miRNA and the CUL4B and HBx expression were detected by Western blot. Error bars represent mean ± *SD* of three independent experiments. (**$P < 0.01$; ***$P < 0.001$).

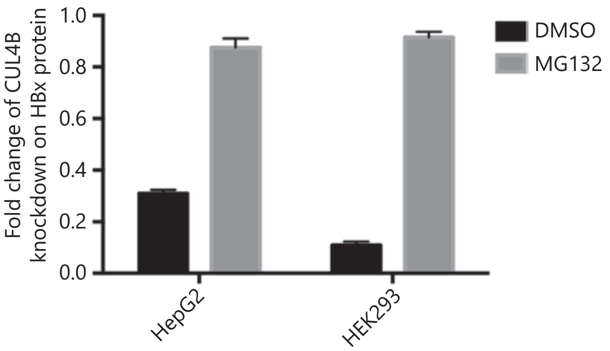

Figure S6 Related to **Figure 5**: CUL4B inhibits the ubiquitination and proteasomal degradation of HBx. The band intensity in Western blot assay in **Figure 4A–D** by gray scanning analysis. Fold change of CUL4B knockdown on HBx protein level was calculated in DMSO- or MG132-treated group relative to Neg-miRNA controls in HepG2 and HEK293 cells. Error bars represent mean ± *SD* of three independent experiments.
